# Controversies and evidence on Chlamydia testing and treatment in asymptomatic women and men who have sex with men: a narrative review

**DOI:** 10.1186/s12879-022-07171-2

**Published:** 2022-03-14

**Authors:** Nicole H. T. M. Dukers-Muijrers, Ymke J. Evers, Christian J. P. A. Hoebe, Petra F. G. Wolffs, Henry J. C. de Vries, Bernice Hoenderboom, Marianne A. B. van der Sande, Janneke Heijne, Jeffrey D. Klausner, Jane S. Hocking, Jan van Bergen

**Affiliations:** 1 Department of Sexual Health, Infectious Diseases, and Environmental Health, South Limburg Public Health Service, PO Box 33, 6400 AA Heerlen, The Netherlands; 2 Department of Health Promotion, Care and Public Health Research Institute (CAPHRI), Maastricht University Medical Center (MUMC+), Maastricht, The Netherlands; 3 Department of Social Medicine, Care and Public Health Research Institute (CAPHRI), Maastricht University Medical Center (MUMC+), Maastricht, The Netherlands; 4 Department of Medical Microbiology, Care and Public Health Research Institute (CAPHRI), Maastricht University Medical Center (MUMC+), Maastricht, The Netherlands; 5 Epidemiology and Surveillance Unit, Centre for Infectious Disease Control, National Institute of Public Health and the Environment (RIVM), Bilthoven, The Netherlands; 6 Department of Infectious Diseases, Public Health Service of Amsterdam (GGD Amsterdam), Amsterdam, The Netherlands; 7 Department of Dermatology, Amsterdam Infection & Immunity Institute (AII), Amsterdam University Medical Center (UMC), Amsterdam, The Netherlands; 8 Institute for Public Health Genomics, Genetics & Cell Biology, Maastricht University, Faculty of Health and Medicine and Life Sciences, Maastricht, The Netherlands; 9 Department of Public Health, Institute of Tropical Medicine, Antwerp, Belgium; 10 Global Health, Julius Centre for Health Sciences and Primary Care, UMC Utrecht, Utrecht University, Utrecht, The Netherlands; 11 Department of Population and Public Health Sciences, Keck School of Medicine of the University of Southern California, Los Angeles, USA; 12 Melbourne School of Population and Global Health, University of Melbourne, Parkville, Victoria, Australia; 13 Department of General Practice, Amsterdam UMC, University of Amsterdam, Amsterdam, The Netherlands; 14 STI AIDS Netherlands, Amsterdam, The Netherlands

**Keywords:** Testing, Treatment, Urogenital, Pharyngeal, Rectal, Extragenital, *Chlamydia trachomatis*, Women, Men who have sex with men

## Abstract

**Background:**

*Chlamydia trachomatis* (CT) is the most common bacterial sexually transmitted infection (STI) worldwide. CT is mainly asymptomatic. Test-and-treat strategies are widely implemented to prevent transmission and complications. Strategies are not without controversy in asymptomatic women and men who have sex with men (MSM). Concerns are emerging to test and treat asymptomatic persons for urogenital CT (‘Controversy 1’) and pharyngeal or rectal CT (‘Controversy 2’), whereby testing symptomatic persons is not under debate. Opposed views in CT treatment involve using azithromycin versus doxycycline (‘Controversy 3’). The objective of this review is to provide coverage of these public health and clinical controversies by reviewing the current scientific evidence.

**Methods:**

A literature search was performed using PubMed for relevant publications between 2018 and September 2021, and iterative retrieval of additional relevant publications.

**Results:**

Controversy 1. In women, the majority of asymptomatic CT are at the urogenital site, and detections mostly include viable CT. CT easily transmits to a partner and potentially also between the vaginal and rectal areas; the clinical impact of urogenital CT is established, although risks for adverse outcomes are uncertain. Wide-scale testing in asymptomatic women has not resulted in reduced prevalence. In MSM, evidence for the clinical impact of asymptomatic urogenital CT is lacking. Controversy 2. Rectal CT is common in women diagnosed with urogenital CT, but the clinical impact of asymptomatic rectal CT is uncertain. In MSM, rectal CT is common, and most CT infections are at the rectal site, yet the risk of longer term complications is unknown. In both sexes, pharyngeal CT is uncommon and has no documented clinical impact. Controversy 3. In the treatment of rectal CT, doxycycline has superior effectiveness to azithromycin. Evidence has also accumulated on the harms of test-and-treat strategies.

**Conclusions:**

Current practices vary widely, from widescale test-and-treat approaches to more individual patient- and partner-level case management. Choosing which asymptomatic people to test at what anatomic site, and whether to test or not, requires an urgent (re-)definition of the goals of testing and treating asymptomatic persons. Treatment guidelines are shifting toward universal doxycycline use, and clinical practice now faces the challenge of implementation.

**Supplementary Information:**

The online version contains supplementary material available at 10.1186/s12879-022-07171-2.

## Short summary

Test-and-treat is a key strategy in the control of Chlamydia trachomatis (CT). We discuss recent controversies and present scientific evidence regarding urogenital, rectal, and pharyngeal CT test-and-treat strategies in women and in men who have sex with men (MSM). This should inform best practices for the prevention and management of the most common bacterial sexually transmitted infection (STI) worldwide.

**Supplementary Information:**

The online version contains supplementary material available at 10.1186/s12879-022-07171-2.

## Background

*Chlamydia trachomatis* (CT) is the most reported bacterial sexually transmitted infection (STI) in the world, with 406,406 confirmed cases of CT infection in the 26 EU/EEA Member States in 2018 [1, 2]. Most cases are detected in young people below 25 years of age. The initial infection is often asymptomatic but might include vaginal discharge [3]; asymptomatic infection is only revealed by active testing. If left untreated, infection can result in pelvic inflammatory disease (PID), ectopic pregnancy (EP), and tubal infertility (TFI) in women [4, 5]. CT can be reliably tested on self-collected samples using nucleic acid amplification tests (NAATs) and is treated with azithromycin (1 g orally) as a single dose, or with doxycycline (100 mg orally twice a day for seven days), depending on the anatomic site of infection and local STI guidelines.

CT control strategies aim to prevent transmission and complications in the population and the individual. In men who have sex with men (MSM), the main aim is to accurately detect and treat symptomatic CT, including lymphogranuloma venereum (LGV), and to prevent the onward transmission of this more pathogenic strain. In MSM, the potential value of testing asymptomatics is also that chlamydia control will help to reduce the risk of HIV acquisition and transmission.

**Fig. 1:**
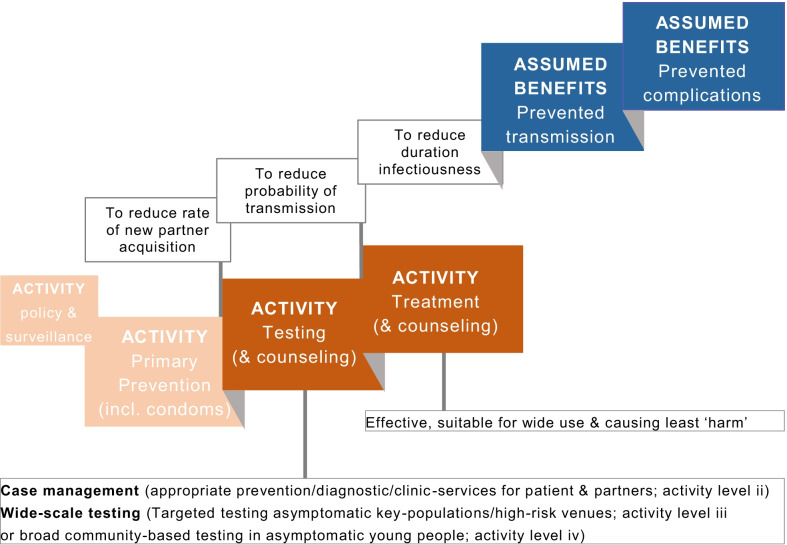
Chlamydia control cascade with f control activities, individual and population assumed and desired program benefits

To develop and implement CT control activities, guidance from the European Centre for Disease Prevention and Control suggests a framework, a CT control cascade, that public health authorities can adopt (Fig. 1). Aside from the integral activity of implementation and surveillance, four levels of incremental activity are recommended [1]. These are level (i), primary prevention (including behavioural counselling; promoting sexual health, safer sex, condom use; and reducing the partner change rate) to reduce transmission efficiency; level, (ii) case management (appropriate diagnostic, clinical, and partner notification services); level (iii) opportunistic/targeted testing in key populations (e.g., those visiting certain venues such as STI clinics or housed in jails or congregating in schools); and level iv) organized community-based testing/screening (e.g., young women) [1]. Test-and-treat strategies aim to reduce the duration of infectiousness, thereby also reducing subsequent complications and future transmission (Fig. 1). See Table 1 for current guidelines in women and MSM [6–9].

**Table 1:** Guidelines on testing and treatment of uncomplicated chlamydia (CT) in asymptomatic women and MSM

Testing and treatment^^^	Urogenital CT	Pharyngeal CT	Rectal CT
Europe (6), UK (7), Australia (9), US (8)	Routine testing (or when a change of sex partner occurs) in people < 25 or 30 years of age and in key populations; implementation in countries varies (e.g., age-based, or by behavioural risk, specific settings/venues) in women and MSM	No testing or selective testing based on reports of oral sex or symptoms in women at STI clinics. Several countries recommend routine testing in MSM	No testing or selective testing based on reports of anal sex or symptoms in women at STI clinics. Most countries recommend routine testing in MSM
Treatment*			
Europe (‘15)^#^	Azithromycin	Azithromycin	Doxycycline
UK (‘18)	Doxycycline	Doxycycline	Doxycycline
Australia^#^	Doxycycline or Azithromycin	Doxycycline or Azithromycin	Doxycycline
USA (2021)	Doxycycline	Doxycycline	Doxycycline

^^^Concerns opportunistic/targeted testing. In addition, in some countries, young people could/can enter community-based testing programs

*Recommended first-line treatment shown

^#^In the progress of revising guidance

This narrative review highlights three relevant controversies about test-and-treat strategies for asymptomatic chlamydial infection and will explicitly cover two populations: women and MSM. These controversies express opposing views: (1) large-scale or targeted testing or limited urogenital testing of asymptomatic persons; and (2) routine testing or limited testing of asymptomatic persons for pharyngeal and rectal CT. Currently, we see a great variety of strategies applied in practice and in various populations. Most countries have installed targeted testing of key populations in STI clinics. Some countries are newly starting wide-scale screening in primary care (e.g., general practice, community health, family practice), while other countries are shifting their focus toward improving case management, with an eye on the patient and partners. There has long been controversy regarding the best treatment choice, but recent treatment guidance is moving toward more uniform recommendations. The controversy on treatment is currently most visible in practice; that is, whether to (3) treat CT patients with azithromycin or with doxycycline for urogenital and pharyngeal infections. These controversies and their supporting arguments are outlined in Table 2.

**Table 2:** Opposing views and arguments on the current testing and treatment strategies in chlamydia (CT) control

Controversy	Main view and raised arguments	Main view and raised arguments
1. Urogenital testing	Widely implement testing in asymptomatic key populations/communities	Reduce testing of asymptomatic women and MSM
	1.1. In women, urogenital CT is prevalent, easily transmitted, and may cause complications	1.3. Test implementation in ‘real-life’ does not achieve the desired benefits (of reducing prevalence and avoiding complications)
	1.2.With resources available, CT is easy to test	1.4.Testing may also bring harm
2. Extragenital testing	Test more to reveal missed extragenital infections	Reduce testing of asymptomatic CT with limited ‘relevance’
	2.1. CT can occur at the pharyngeal and rectal sites in women and MSM	2.3. Rectal CT might not always reflect a ‘true’ infection in women
	2.2. Rectal CT may comprise a ‘hidden’ reservoir of transmissible infections in women and MSM, and increase the risk for HIV acquisition and transmission in MSM	2.4. Clinical impact of pharyngeal or rectal (non-LGV) CT may be limited
3. Treatment	Use azithromycin	Use doxycycline
	3.1. Azithromycin is easy to use, safe, and widely applicable	3.3. The risk of azithromycin treatment failure is high in rectal CT
	3.2. Azithromycin is effective in curing urogenital and pharyngeal CT	3.4. Treatment, especially azithromycin, can cause AMR

*The primary objective of this paper* is to provide substantial coverage of the main controversies in these current clinical and public health areas, i.e. urogenital, pharyngeal, and rectal testing and treatment of asymptomatic women and MSM (Fig. 1). This review thereby adds to the existing literature on testing and treatment, placing these issues in the wider context of the CT control cascade in women and in MSM. This review includes state-of-the-art research, reviews (which are usually more narrowly focused), and opinion papers (which are usually less complete regarding scientific detail) on test-and-treat strategies. As for the controversies surrounding testing, the themes outlined include the potential for the onward transmission of asymptomatic CT and for causing complications, the realistically achievable benefits (avoided transmission and complications), and possible harm induced by testing. In terms of the controversies about treatment, the themes outlined include effectiveness and applicability, and possible harm induced by treating asymptomatic CT infection. We close with a summary and reflections on resolving the controversies related to testing and treatment to inform CT control.

## Methods

**Fig. 2:**
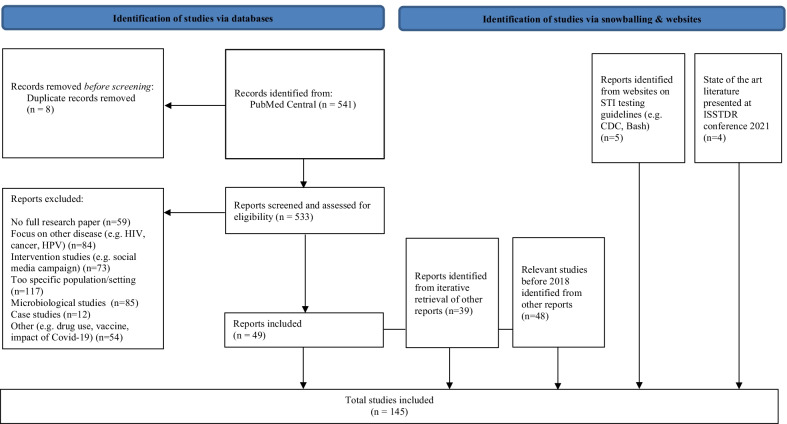
Flowchart of the retrieved literature

We (the authors ND and YE) searched PubMed for publications; see the flowchart for the retrieval of the publications (Fig. 2). Since we searched for newer studies that add to the body of existing literature, we started with the most recent papers, namely, from 2018 to June 2021 (Additional file 1). ND and YE assessed the retrieved abstracts subjectively for novelty and study quality, and, based on the expert opinions of all co-authors, we selected the most relevant papers. Further, all co-authors iteratively retrieved pertinent referenced papers, including those published before 2018. In addition, we included relevant state-of-the-art published ISSTDR conference abstracts (July 2021); we updated these abstract-references with the peer-reviewed papers on these abstracts, when these papers were published between the literature retrieval and the publication of this review. All authors contributed to the synthesis and interpretation of the literature.

## Results

### Controversy 1: Testing for urogenital chlamydia

Over time, particularly in high-income countries, discussions about CT control have come to focus more on widescale testing for asymptomatic CT [10], and routine urogenital CT testing is advocated in young women and in most guidelines for MSM [6–9]. This prevailing view is primarily based on the arguments that (1.1) ‘in women, urogenital CT is prevalent, easily transmitted, and may cause complications’; and (1.2) ‘with resources available, CT is simple to test and treat in women and MSM’. In 2017, Unemo et al. [10] set the stage for rethinking the testing of asymptomatic persons. With regard to the CT control cascade, challenges were identified, including setting realistic targets for the achievable benefits of test-and-treat strategies, and pleas were made to focus more on monitoring health outcomes and preventing complications, such as PID in women (rather than preventing infections), with a focus on improving case management for the patient and his/her sex partners (CT control activity level ii). In line with these ideas, a narrative review [11] expressed concerns on the widescale testing of asymptomatic women and MSM based on the arguments that (1.3) ‘test implementation in “real-life” does not achieve the desired benefits’ and (1.4) ‘testing may also bring harm’.

#### In women, urogenital CT is prevalent, easily transmitted, and may cause complications.

*Prevalence in women* The prevalence of urogenital CT in women worldwide was estimated to be approximately 3% in two meta-analyses [12, 13]. Prevalence estimates (range: 0.2–12.2%) vary substantially by geography on both the large-spatial scale and the smaller local scale [12, 13], reflecting the relatively high impacts of several social determinants of health such as socioeconomics, demographic characteristics, social vulnerability, and access to care. Chlamydia testing positivity is higher at venues such as STI clinics, emergency departments, youth homeless shelters, and among populations historically disadvantaged by structural and persistent racism. It should be noted that positivity in tested populations does not directly reflect population prevalence [14].

*Bacterial load* The CT bacterial load may differ between tested populations, possibly reflecting different periods of having an infection before being tested [15, 16]. In Dutch CT-infected women, primary care patients had a higher mean urogenital CT load than STI clinic patients, while hospital (e.g. gynaecology) or community-based tested patients had lower mean load [15, 16]. Data in women in a US study [17] and laboratory registry data in men and women in a Dutch study [18] and in Australian women [19] showed that urogenital CT load was lower in repeat infections than in initial CT infections. Proposed hypotheses for this phenomenon [20] include that past infection may confer some protective immunity and impacts on organism replication (but not chlamydial entry), or that initial CT infection in tested populations may represent a biased sample of higher load infections that have not cleared (as lower load infections clear more quickly). Large-scale epidemiological evaluations of CT load have become feasible by using the cycle quantification value of the NAAT test as a proxy as a practical method aiding in CT epidemiology. Various other, more laborious methods have been used, and a review revealed 14 different methods in 28 studies, severely hampering comparison, and calling for the standardization of load measurement and reporting [21]. It is of interest to learn about associated factors for CT load, but only a few factors (e.g. young age) were observed in men and women [16, 17, 20, 21].

*Spontaneous clearance in women* Most asymptomatic CT infections will resolve spontaneously if not treated, and the median time to natural clearance for urogenital CT is approximately one year [22, 23]. The spontaneous clearance of urogenital CT between diagnosis and treatment (9–10 days) was 6–9% in large-scale evaluations of urine samples and vaginal swabs, and was faster with a lower baseline bacterial load [24, 25]. In women, spontaneous clearance was high (i.e. 32%) when vaginal CT was a single anatomic site infection, possibly indicating low load detections. Between diagnosis and treatment, vaginal CT rarely cleared (2%) when rectal CT was initially diagnosed [25].

*Viability in women* A limitation of NAAT is that it cannot distinguish between viable bacteria and non-viable molecular remnants. New assays were recently developed to measure CT viability in clinical samples; that is, via the detection of messenger RNA by digital PCR [26], and the use of V-PCR by Australian and Dutch laboratories [27]. These assays are highly sensitive in contrast to cultures, but also laborious and thus applied in research contexts only. The presence of viable organisms does not prove—yet strongly indicates—that the NAAT-detected CT is a ‘true infection’. In nearly all digital PCR/V-PCR tested vaginal samples, viable CT was detected Of CT-DNA-positive vaginal samples, 83% (24/29) had mRNA detected [26], and 94% (469/499) showed viable CT by V-PCR in a prospective cohort study [28]. Women had lower viability of vaginal CT (with lower viable load) when they did not have a rectal CT at the same time. Vaginal CT viability was 48% in single-site vaginal CT [25]. The vaginal viable load (by V-PCR [27]) was slightly higher with symptoms of altered vaginal discharge, although almost half of the women in the highest viable load quartile did not have symptoms in a prospective cohort study [28].

*Transmission between women and their male sex partners* Chlamydia transmits easily. Transmission probabilities were estimated by modelling at 2–15% per sex act in heterosexual, 32–35% per partnership from men to women, and 5–21% from women to men in a UK modelling study [29]. It is posited that the transmission between partners increases with high organism load, although prospective data are lacking. A US cross-sectional study of heterosexual couples showed that CT-infected women with a CT-positive male partner had a higher median load than women with a CT-negative partner, although causality is unknown [30].

*Transmission between anatomic sites within a woman* Transmission occurs between persons and possibly also between the vaginal and rectal sites in women. Evidence for autoinoculation has been provided in a mathematical model in women attending STI clinics, with a daily probability of 0.5–1% that a urogenital infection leads to a rectal infection, or vice versa [31]. This was also supported by prospective observational data (15–40 two-week follow-up periods) showing that urogenital CT, especially with high load, was strongly predictive of rectal CT acquisition two weeks later without sexual exposure [32].

*Complications in women* In women, CT infections can initiate inflammatory and immunological processes leading to several reproductive complications, such as PID, which can lead to chronic abdominal pain, EP, and TFI [33, 34]. Repeat infection increases the risk of PID [34]. In a statistical evidence synthesis, the authors estimated that every 1000 CT infections led to 171 episodes of PID, 73 cases of salpingitis, 2 EP, and 5 TFI [35]. Prospective Dutch data showed a slight delay in time to pregnancy [36]. Other complications include adverse pregnancy outcomes such as preterm birth, low birthweight, and postpartum infections [37]. Two meta-analyses confirmed associations with a range of adverse pregnancy outcomes, but also noted the uncertainty of estimated risks due to bias in the design and conduct of studies [38, 39]. Risks of spontaneous abortion, infertility, and ectopic pregnancy appeared higher in low- and middle-income countries than in high-income countries, with unknown reasons [38]. Due to bias in study designs, there is uncertainty in causality inferences and uncertainty in estimated risks, also due to different assessments of confounders and outcome definitions [40]. This makes it very difficult to know the actual risks and preventable fractions.

*Urogenital CT in MSM* The arguments for testing urogenital CT in clinical and public health practice are mainly based on the epidemiology in women. MSM, in whom urogenital CT testing also is routinely recommended in international guidelines, generally show a lower urogenital CT positivity than women, in studies in tested clinic populations [41, 42], although prevalence estimates are scarce. Urogenital CT was estimated to be 0.4% in the general MSM population by respondent-driven sampling in Canada [43]. In tested populations in STI clinics, urogenital CT positivity varies but it is generally lower than that established for rectal CT in MSM (see below). Bacterial load in urine samples was found lower than in vaginal samples but comparisons are hampered due to different sample materials [16]. Spontaneous clearance data on urogenital CT in men are scarce [24], though in most mathematical models it was assumed comparable to that of women. Viability data by digital-PCR or V-PCR have not been reported for urogenital CT in MSM. Symptoms of urogenital CT in MSM are uncommon but include urethritis and epididymitis; however, they may also include rare sexually acquired reactive arthritis or perihepatitis.

#### With resources available, CT is easy to test

In women and MSM, the advent of commercial NAATs has driven large increases in testing at multiple anatomic sites and in various populations. These assays diagnose CT, including LGV, detecting all serovars. NAATs are highly sensitive for detecting CT-DNA/RNA in first void urine and self-taken vaginal, rectal, and pharyngeal swabs; self-collection is well and widely accepted in women and in MSM as shown in prospective and retrospective cohort studies [44–48]. A randomized clinical trial (RCT) in women and MSM showed good concordance between clinician-and self-taken pharyngeal and rectal swabs, with similarly high diagnostic accuracy [49]. Using outreach at high-risk venues, combined with counselling [50], new developments in home sampling, Internet testing, and point-of-care testing (same-day treatment, partner packs) all offer locally practical opportunities for implementation [51–54].

#### Test implementation in ‘real-life’ does not achieve the desired benefits

*Women* Mathematical models confirm that widescale testing of asymptomatic women should be effective to reduce the duration of infectiousness [55] and CT prevalence [56–58]. However, pragmatic studies indicate that it might be difficult to achieve a reduction in prevalence and in complications. Low test uptake hampered community-based testing in young people in the Dutch Chlamydia Screening Implementation trial and a cluster RCT in Australia [59, 60]. Sustained uptake of widespread testing was deemed not feasible and unlikely to achieve a sizeable reduction in prevalence. In England, there is an absence of evidence that chlamydia screening has impacted population prevalence [61], even though the National Chlamydia Screening Programme has resulted in a significant increase in STI testing capacity in England [61].

Targeted testing involves offering tests to key populations such as high-risk young women, sex workers, or MSM; for example, in general practice or ‘high-risk settings’ such as emergency departments, homeless shelters, and STI or HIV clinics. A meta-analysis reported that community-based testing in general populations may make little-to-no difference for CT transmission and a woman’s risk of PID or EP; evidence on infertility was very uncertain, and no evidence was found for cervicitis or chronic pelvic pain [4]. Previous studies suggest that screening can reduce PID risk at the individual level [40, 62]. The meta-analysis concluded that benefits might potentially be achieved for reducing CT transmission and PID by targeted and intense (repeat) testing of high-prevalence key female populations [4]. Postulated reasons why test-and-treat strategies might not reduce prevalence in 'real-life' include—alongside low test uptake—that treated patients have lower protective immunity [63], but reasons for the gap between models and practice remain largely unclear.

*MSM* The available data on the impact of testing on CT prevalence reduction in MSM are sparse. An ecological study among MSM in 23 EU countries showed no evidence that testing diminished prevalence based on 2010 data [64]. Large online surveys for MSM (EMIS- ‘10/’17) showed a positive association of country-level testing rates and proportions of symptomatic CT [65]. A review of observational studies in MSM (including 3-month universal testing) did not demonstrate reduced prevalence by test-and-treat strategies [66]. Postulated reasons in MSM include the influx of new infections by untested/untreated male partners.

#### Testing may also bring harm

*Harm in women and MSM* A narrative review stressed that testing may introduce harm that should be carefully weighed against the benefits [11]. Similar concerns have been raised for pharyngeal and rectal CT testing in an editorial [67]. Testing may bring about adverse psychological effects. A meta-analysis reported that undergoing testing or having a diagnosis of CT may cause a small-to-moderate number of people to experience some degree of harm (feelings of stigmatization, anxiety about future infertility, intimate partner violence), with most studies in women [4, 68, 69]. How patients weigh the potential benefits versus the harm of screening was found to be uncertain in this meta-analysis, yet risks to reproductive health (infertility, chronic pelvic pain) and transmission appear to be more important than the (often transient) psychosocial harm involved [4]. However, it is unknown to what extent women over- (or under-) estimated the actual CT complication risks. Furthermore, harms that were not evaluated in this study were those induced by treatment as compromised microbiome, described in a systematic review and metataxonomic analysis [70, 71], antimicrobial resistance (AMR) in *Neisseria gonorrhoea* (NG), *Mycoplasma genitalium* (MG), syphilis, and other pathogenic microorganisms [72–75], as well as a possible arrested immune response [76]. Further ‘harm’ includes economic individual and health care costs (‘value for money’) and issues related to inequity in health care access.

*Benefits versus harms in women and MSM* Recognizing potential harm and the need to balance risks and benefits boosts the rethinking about chlamydia control. Such as what realistic and achievable goals one should strive for, with what types of strategies, and how the focus can be shifted more towards disease control (preventing complications), rather than infection control (preventing infections), as historically championed [10]. Some countries, including low- and middle-income states, are calling for enhanced large-scale CT control, such as targeting socio-spatial high-risk clusters [77]. Some high-income countries are newly starting to recommend large-scale screening in key populations of women under 30 years of age in primary care [78]. However, scientists and physicians are increasingly calling to stop attempting to reduce CT population prevalence through the extensive testing and treatment of asymptomatic women and MSM, and to move the paradigm from infection-control (i.e., test-and-treat to reduce the duration of infectiousness to prevent the infection from spreading) to disease-control (i.e., using strategies specifically to prevent complications) [11, 61], thereby mitigating the harm of test-and-treat strategies.

In line with this thinking, there are attempts to design novel methods for preventing late complications in women by targeting high-risk pathogen and host profiles. In current care, this is complicated because diagnostic tools other than NAAT are unavailable to identify the most ‘infectious’ and ‘pathogenic’ CT, but viability diagnostics may help in the future. To identify those at highest risk of CT, a clear set of risk factors should be used, including PID biomarkers or host immunogenetic factors, which are explored as new avenues for updating existing prediction models for CT-related TFI, as described in narrative reviews [79, 80]. However, these methods still require testing first.

In current practice, the focus is shifting back toward the patient and their partner (CT control activity levels i and ii), rather than remain on asymptomatic communities/key populations (activity levels iii and iv), as called for in a commissioned review paper [10] and evidenced in guidelines (e.g. in Australia) to strengthen primary prevention, and to move toward better case management to reduce the risk of reinfection and of PID through partner management, patient delivered partner therapy, and re-testing at 3 months to detect reinfections early [81]. Case management for women and MSM entails comprehensive sexual health management, history taking, counselling, appropriate diagnostics, clinical examination, clinical care, partner notification, health promotion advice, and follow-ups, and is in combination with primary prevention and surveillance in key populations.

### Controversy 2: Testing for pharyngeal and rectal CT infections

In the last decade, pharyngeal and rectal CT in MSM and in women has become a topic of debate, with substantial variation in practices and guidelines [6–8, 82]. In women, pharyngeal and rectal testing is not universally recommended; it is either discouraged when asymptomatic or selective only, i.e., testing based on the criteria of reported risky behaviour or symptoms. For MSM, several international guidelines recommend testing at all three anatomic sites [6–9]. Views that support pharyngal and rectal testing in women and in MSM are primarily meant to prevent onward transmission, and in MSM, to help prevent HIV. These views are based on the arguments that (2.1) ‘pharyngeal and rectal CT do occur in women and MSM’; and (2.2) ‘untested rectal CT may comprise a ‘hidden’ reservoir of transmissible infections and HIV risk in MSM’. Others argue that testing for pharyngeal and rectal CT has limited benefits, grounded in the arguments that (2.3) ‘rectal CT in women may not always reflect a 'true' infection’, and (2.4) the ‘clinical impact of pharyngeal or rectal (non-LGV-) CT may be limited’.

#### CT can occur at the pharyngeal and rectal sites in women and MSM

*Test positivity, clearance, load, and viability of rectal CT in women* Rectal CT test positivity is high, i.e. 9% in women tested in sexual health clinics and non-sexual health clinics, according to a meta-analysis [83]. In women diagnosed with urogenital CT, rectal CT is common, at approximately 70% [84]. In women, CT LGV is rare except for certain populations (e.g., shown in 20% of female-HIV patients in South Africa) [85], but systematic LGV assessment is lacking in women. Rectal CT is predicted by the presence of a (one’s own) urogenital CT, but is not predicted by anal sex or symptoms in women and MSM [83, 86, 87]. Spontaneous clearance in women between diagnosis and treatment is relatively common at 16–18% [24, 25]. Spontaneous clearance was more common with a lower baseline rectal CT load [88] and in women when rectal CT was a single anatomic site infection [25]. The CT load in rectal samples was lower than that in vaginal samples [16, 89] but comparable between women and MSM [18]. In women, a rectal CT infection often remains untested. In a cohort study of women diagnosed with urogenital chlamydia, 77% (272/351) had rectal CT detected when they returned to the clinic for treatment [25]. Of NAAT-positive rectal samples in women, 66% (290/436) had viable rectal CT by V-PCR, raising the possibility that the remaining 34% of samples were the result of detected CT nucleic acid, but not active 'true' infections. In another study of rectal NAAT-positive women, 60% (6/10) were viable by culture [25, 90]. The cross-sectional evaluation of viable rectal CT load did not reveal associations with reported anal sex or symptoms [28].

*Positivity, clearance, and load of rectal CT in MSM* Positivity is 9% in MSM according to a meta-analysis [83]. Positivity may vary geographically [91]. Rectal CT is mainly asymptomatic, as observed in both STI clinic and community clinic settings in higher- and low-resource environments [92, 93]. A study in MSM estimated the duration of rectal CT at 13 weeks [94]. In MSM, 5–22% of all (symptomatic and asymptomatic) rectal CT is of the CT LGV biovar, although in many assessments, proportions were found at the lower end of this range [42]. Only some laboratories routinely test all rectal positive samples for LGV. In MSM, the rectal site is the main site of all CT infections, as demonstrated in clinic based studies [86, 87, 95]. Spontaneous clearance in MSM between diagnosis and treatment was 4–18% [24, 88]. The bacterial load is comparable to that of rectal CT load in women [18].

*Positivity, clearance, load, and viability of pharyngeal CT in women and MSM* Pharyngeal CT test positivity is between 1 and 3% in women and in MSM in clinical and non-clinical venues [89, 95–99]. Pharyngeal CT is not associated with reported oral sex. The spontaneous clearance between diagnosis and treatment is high: 36–57% (interval: 9–10 days) in men and women [24, 32, 89]. A study in MSM in Seattle estimated the median duration of pharyngeal CT at 6 weeks [100]. Spontaneous clearance is more common with low baseline bacterial load and in a single anatomic site CT, as is also established in urogenital and rectal CT [24, 25, 32, 89]. The pharyngeal CT load is low and similar between men and women [18]. In MSM with pharyngeal CT, an Australian prospective cohort study showed that 69% (29/42) had CT-DNA in saliva, but with unknown viability [101]. Viability was examined in women, and 26% (12/46) had viable pharyngeal CT by V-PCR;  in comparison, it was 94% in vaginal and 66% in rectal CT [25, 102]. In a substantial proportion of pharyngeal detections, non-viable CT may possibly reflect non-viable molecular nucleic acid remnants rather than a 'true' pharyngeal infection.

#### Untested rectal CT may comprise a ‘hidden’ reservoir of transmissible infections in women and MSM, and increase the risk for HIV acquisition and transmission in MSM

*The number of CT missed in different testing scenarios in women and MSM* The number of untested asymptomatic pharyngeal and rectal infections in regular STI care depends on test-and-treat strategies in terms of place, the type of population served, and epidemiological and geo-sociodemographic aspects. Nevertheless, some general conclusions can be drawn from studies in multisite tested populations. Single-site pharyngeal and rectal CT infections will per facto remain undetected if only urogenital CT is tested, as is most often the case in women. Studies of women from STI clinics have shown that 5–20% of rectal chlamydia detections are single anatomic site infections [103–105]. Of all rectal CT in men, 80–85% are single anatomic site infections, as demonstrated by evaluations in all Dutch STI clinics [86, 87, 95]. Of all pharyngeal CT, 20% are single anatomic site infections in women and 40% in MSM [89, 95–97].

In women, up to 25% of all CT are single anatomic site CT infections, as revealed by a cross-sectional study that applied multisite testing, e.g. in women attending a community health centre in the US [106], in women who attended an STI clinic in Italy [104], and in a large group of sexually active young adults in the US [103]. In MSM, a higher proportion of all CT infections than in women, are single infections; 63–80% of all CT in MSM are single non-urogenital anatomic site CT infections [47, 103, 107–109]. To give two examples, in a US prospective cohort study of all CT, 68% were rectal only, 6% rectal and pharyngeal, 5% pharyngeal only, and 13% urogenital only [103]. In a Dutch study of all CT 60% were rectal only, 6% rectal and pharyngeal, 4% pharyngeal only, and 19% urogenital only [105].

*Impact of missed CT estimates on STI clinical populations, in women and MSM* To understand what these distributions mean in terms of actual CT infections missed, we need to consider CT test positivity in the source population. To give a simple calculation as an illustration, given a pharyngeal CT test positivity of 1–3%, of which 20–40% is single-site pharyngeal CT [96, 98] translates to only 0.2–1.2% of women and MSM in clinical care practice who have a single-site pharyngeal CT. Given a rectal CT test positivity of 9%, of which 20% (in women) or 80% (in MSM) is single-site rectal CT, translates to only 1-2% of women who have a single-site rectal CT. In MSM, this is higher at 7% of MSM. Indeed, in an (n = 498 CT) Australian evaluation, 8% of MSM clinic attendees showed single-site rectal CT, while 0.7% had single-site pharyngeal CT, and 2% had single-site urogenital CT [108]. As explained before, single anatomic site infections will likely spontaneously clear faster, and part of the detected single anatomic site CT will be non-viable (e.g. data in women show that 74% of pharyngeal and 34% of rectal CT are non-viable [25, 28, 102]).

*Pharyngeal and rectal testing-practices in women and MSM* Three-site testing (urogenital, anorectal, and pharyngeal) is recommended in MSM attending STI clinics in various countries. Another scenario would be two-site anorectal and pharyngeal testing. A prospective cohort study among young MSM aged indicated that this two-site testing scenario would detect 93% of CT infections [103]. Other scenarios are selective testing scenarios, based on reports of anal or oral sex. These have been used to reduce missed infections without having to test all people at all anatomic sites. However, selective testing still misses approximately 50–75% of all rectal and pharyngeal CT in both women and MSM [96, 98, 107]. There have been attempts to optimise selective testing criteria on ‘who-to-test’. In a large-scale Dutch study based on STI clinical data in routinely tested MSM (1% pharyngeal CT; 2041/176,895) and women (2% pharyngeal CT; 1081/45,774), varying the criteria for testing failed to optimise the performance of selective pharyngeal testing strategies [95, 98]. Likewise, attempts to explore algorithms for selective rectal CT testing based on a priori patient characteristics were unsuccessful, yielding low discriminatory power [87]. Another, more economic scenario is to choose the anatomic site that would reveal the most CT infections through one-site testing. In MSM, one-site rectal testing and one-site urogenital testing in women detect approximately 80% of all CT infections in MSM and in women [103, 105, 107]. Importantly, in women, one-site urogenital testing with subsequent treatment also allows for co-treating concurrent rectal CT (co-treating the main share of all rectal CT in women), thereby enabling more comprehensive CT management. Another scenario is pooling samples self-collected from various anatomic sites (e.g. urogenital, rectal, or pharyngeal swabs) or gargle material [110]. A large UK randomized controlled trial confirmed that pooling was only slightly less sensitive in detecting infections than three-site testing and at lower costs [49].

*Transmission of rectal CT in women and MSM* Viable CT at the rectal and pharyngeal site is likely transmissible to a sex partner. An Australian modelling study verified that the transmission of rectal and pharyngeal CT in MSM is not singularly limited to anal or oral sex, and that single contributions of any sexual practices are hard to disentangle [111, 112]. In MSM, it is estimated that penile-anal sex contributes most to CT transmission. In women, it is likely that rectal CT can be transmitted to a sexual partner given the increasing reports of heterosexual anal sex, although heterosexual transmission of rectal CT was not studied. In women, there is an ongoing debate about the possibility of transmission between anatomic sites within a person. A Dutch modelling study estimated that more rectal CT in women who visited STI clinics was caused by autoinoculation from the urogenital to the rectal location than by anal sex [31]. Observational data also suggest that rectal CT may migrate between the rectal and vaginal site, by an autoinoculation process [32]. Women who reported intercourse or other sexual exposure risks (but who had not had a preceding rectal CT) had a urogenital 2-week incidence of 2%; this incidence was higher in women who had a preceding rectal CT (14% when they did not report sex, and 25% when they reported it) [32]. Without the appropriate management of rectal CT, we can thereby hypothesise that rectal CT in women may comprise a ‘hidden’ reservoir of transmittable infections and play a role in sustaining CT, in line with an Australian modelling study [113]. However, such a potential contribution of rectal CT in further CT transmission and CT-related reproductive complications, is unquantified. In women, only scarce data are available on single-site rectal CT. While viability and viable load were comparable in single-site vs. concurrent rectal CT [27], single-site rectal CT is less prevalent and more likely to spontaneously clear than concurrent rectal CT [25]. This may suggest that the contribution of single-site rectal CT in women to CT transmission is low.

*Transmission of pharyngeal CT in women and MSM* In both, the contribution of pharyngeal CT to total CT transmission is very small [100, 112]. Findings in mice, in which pharyngeal CT may pass the gastrointestinal tract to the rectal site, are not evidenced by human data. Prospective epidemiological studies in women and MSM did not show an independent association between pharyngeal CT and a subsequent rectal CT [25, 32, 114–117].

#### Rectal CT may not always reflect a ‘true' infection in women

Rectal infection with chlamydia is initiated higher up the anorectal canal at the anal verge. However, the detection of rectal chlamydia is usually by NAAT on self-collected samples at the lower rectal site and taken to represent the active ‘true' infection that is higher up the anorectal canal. However, there is concern that such NAAT detection might not always represent a 'true' infection. This concern was raised for rectal CT in women but not in MSM, even though they demonstrate similar rectal CT prevalence and bacterial load, and there is no association with report of recent anal sex [84]. One important difference is that in women, rectal CT mostly co-occurs with urogenital CT, which gave rise to an alternative hypothesis for the detection of rectal CT. One is that CT-DNA is detected from contamination, e.g. from vaginal fluid seepage or transient CT-DNA from semen during sex. When following this reasoning, self-collected rectal samples would not reflect the situation at the actual site of infection. Unique data from a Dutch study in women who attended an STI clinic do not support this argument [118]. Nurse sampling in the lower rectal area and proctoscopy sampling in the columnar cells higher up the anorectal canal showed that when the lower area was NAAT positive (n = 11) or had viable CT (n = 8), the rectal sample higher up was also positive and had viable CT. Higher up the anorectal canal, positivity, viability, and mean viable load were even higher, suggesting that (viable) rectal CT may be underestimated by self-sampling at the rectal site. However, it cannot be ruled out that viable CT may have been detected that has not infected tissue. Further data to inform this discussion are expected from a Swedish prospective cohort study (clinicaltrials.gov/NCT04030949). Notwithstanding, current data do not corroborate concerns that a rectal CT diagnosis in women would not represent a ‘true' rectal infection; this is irrespective of how the rectal CT ‘got there’.

#### The clinical impact of rectal or pharyngeal (non-LGV) CT may be limited

*Complications of rectal CT in women and MSM* Pharyngeal or rectal CT infections are mostly asymptomatic. Rectal CT can cause proctitis in MSM and might be of the LGV biovar. LGV is more often symptomatic and, when untreated, can lead to sequelae, such as anorectal fistulae. The risk of late complications by pharyngeal or rectal non-LGV CT is unknown but probably limited. In settings where PrEP use is not widespread, rectal CT might double the risk of HIV acquisition if the HIV-infected index person is not virologically suppressed according to a meta-analysis, although the evidence is not strong as based on observational data, with inherent confounding factors that may play a role [119, 120]. In women, evidence on the clinical impact of rectal CT is lacking. A possible impact on reproductive complications (via autoinoculation) is speculative and unquantified; rectal sequalae were not described in non-LGV CT.

*Complications of pharyngeal CT* are absent in MSM and in women.

*Benefits of testing versus harm in women and MSM* The Dutch multidisciplinary STI guidance committee [121] previously concluded that pharyngeal CT should not be tested in women or men, including MSM. Nevertheless, pharyngeal CT testing in MSM is routine practice in various settings because of dual testing with NG, as in Dutch STI clinics [95], and testing is recommended in various international guidelines [6]. In an editorial [67], experts stressed that routine pharyngeal and rectal testing of asymptomatic persons must be considered in terms of the clinical data that demonstrate benefit to the individual of the early detection and treatment of those infections, and to public health showing that early detection and treatment reduces the community prevalence and spread of infection. To substantiate these concerns requires careful consideration of which gains are desired and realistically achievable from (extra)genital testing specific populations.

### Controversy 3: Treatment in chlamydia

Ideally, treatment is effective with a microbiological cure rate of at least 95% and is easy to take; one-day treatment has a low side-effect profile and causes minimal interference with one’s daily lifestyle. Views in favour of using azithromycin are based on the arguments that (3.1) ‘the single dose azithromycin is easy to use’ and (3.2) ‘effective in curing urogenital and pharyngeal CT’. Although azithromycin is used widely in practice, recent guidance is shifting toward advocating for the use of doxycycline because (3.3) ‘azithromycin failure risk is high in rectal CT’ and (3.4) ‘treatment, especially azithromycin may induce AMR for non-CT pathogens’.

#### Azithromycin is easy to use, safe, and widely applicable

Single-dose azithromycin has been used worldwide as a first-line treatment in CT for over 20 years, has no adherence issues, and side effects are minor gastrointestinal upset, including nausea, diarrhoea and vomiting.

#### Azithromycin is effective in curing urogenital and pharyngeal CT

In women and men, a meta-analysis of RCTs comparing treatments in urogenital CT showed that the microbiological failure risk was low, with 8 per 100 for azithromycin and slightly lower (3 per 100) for doxycycline in men, and 2 and 1 per 100 in women [3, 122]. A meta-analyses established a pooled azithromycin treatment failure rate of 11% [123]. For pharyngeal CT, there are no controlled studies. Two observational studies assessing pharyngeal CT (in mainly women) revealed microbiological cures for azithromycin of 90% (70/78) and 94% (n = 15/16), and microbiological cures for doxycycline of 98% (63/64) and 100% (n = 20/20) [102, 124].

#### The azithromycin treatment failure risk is high in rectal CT

*Treatment effectiveness in women and MSM* Effective treatment is essential to shorten the infectious period to reduce the risk of transmission and the development of complications (if treatment is in time). Previous observational studies reported the effectiveness of azithromycin in rectal CT to be 83% in MSM in a meta-analysis [125]. In 2021, novel data from the first RCTs were reported. A randomized, double-blind, placebo-controlled trial in MSM in Seattle and Boston indicated microbiological cures of 74% for azithromycin (48/65) and 100% for doxycycline (70/70) [126]. A small number of LGV biovars (4 in each arm) also showed similar cure proportions (i.e. 75% and 100%). Another randomized, double-blind, double-dummy controlled trial in MSM in Australia showed 76% (227/297; 95% CI 74–79) for azithromycin and 97% (281/290; 95% CI 95–99) for doxycycline [127]. The results from the first controlled trial in women, the are underway [128]. Currently, in women only observational data are available, yet highly similar results are shown for women and MSM. The FemCure observational study [116] showed microbiological cure in azithromycin-treated women of 79% (164/209; 95% CI 73–84) and 96% (126/132; 95% CI 91–98) in doxycycline-treated women. With these recent data, there is now strong and consistent evidence that doxycycline is more effective and superior to azithromycin for treating rectal CT in MSM and, with indirect evidence from observational data, for women as well.

*Characteristics of azithromycin treatment failure in women and MSM* Treatment effectiveness was usually assessed by the NAAT result at week 4 [116, 125, 126, 128]. Positive outcomes, in the absence of reinfection risk, indicate treatment failure. As NAAT detects both viable and non-viable organisms, it is important to explore viability in treatment failure. Applying V-PCR to rectal CT failure samples (i.e. taken at week 4 post-azithromycin) in women confirmed high viability [116, 129].

Additionally, the individual time pattern of CT detection has been the subject of interest. So-called (transient negative) ‘blips’ were observed in studies that sampled multiple times after azithromycin was administered to MSM [125] and women [90, 116]. For example, treated MSM exhibited a higher proportion of CT negative samples at week 2 than week 4, and 14% (8/56) of those who were CT negative after 2 weeks were CT positive at week 4 [125]; treated women with CT at week 4 were negative at week 1 or 2 in 58% (26/45) [116]. This pattern of NAAT clearance is opposite to that expected with progressive bacterial clearance, as seen with doxycycline. It was suggested that ‘blips’ might be the detection of non-viable CT in the rectum or perianal regions [90]. However, of women in FemCure who had treatment failure with ‘blips’, 57% had viable CT at the rectal site at week 4 (of failures without ‘blips’, this was 100%) [129]. Another hypothesis to explain ‘blips’ is that the initial rectal CT infection cleared from the columnar cells in the anorectal canal but remained in the upper gastrointestinal tract, resulting in periodic shedding [130], as described in animals; in humans, it is unknown whether CT can establish an infection in the upper gastrointestinal tract.

In assessing factors that could predict azithromycin treatment failure, it was consistently shown that a higher baseline rectal CT load was associated with azithromycin treatment failure in MSM and in women [116, 127, 131, 132]. In women, it was additionally shown that baseline viable rectal CT was key to later viable CT treatment failure [129]. Failure is not associated with sex, baseline report of anal symptoms or anal sex, or other factors that could be useful to guide treatment choice [116, 126, 127]. It is unknown whether antibiotic concentrations are sufficient to cure high pretreatment CT loads, and it is posited that larger or longer doses of azithromycin may be more effective at clearing higher load rectal infections. There is no evidence that CT-related antibiotic resistance or the prevalence of LGV biovars would play a role. A review [133] concluded that it is unclear whether bioavailability, drug solubility, protein binding, the distribution of a drug in intracellular versus extracellular compartments, or local immune response would play a role, and why this should selectively affect azithromycin and not doxycycline.

#### Treatment: especially azithromycin—can also cause anti-microbial resistance (AMR)

*Azithromycin effectiveness, applicability, and harm in women and MSM* While the one-dose regimen enables wide applicability, there are drawbacks such as low effectiveness in rectal CT, and that azithromycin may have a severe impact on AMR prevalence in non-CT pathogenic microorganisms, especially in the dose to treat CT. There is evidence (although inconsistent) that widespread azithromycin use is associated with the development of reduced susceptibility in NG patients [134–136] and selects for macrolide resistance in MG [137, 138], and may play a role in Shigella [138]. Therefore, US guidelines recommend adding doxycycline to the routine treatment of uncomplicated NG if CT has not been excluded [8], and US and UK and guidelines suggest not using azithromycin in CT infection as first line treatment, irrespective of sex or anatomic site of CT infection. Another possible issue with azithromycin (as also with doxycycline) is that early treatment might hamper the immune response in urogenital CT [76]. Azithromycin may furthermore compromise the microbiome, as does doxycycline [70, 71, 139].

*Doxycycline effectiveness, applicability, and harm in women and MSM* Doxycycline is highly effective in resolving CT infection at all anatomic sites, but its multiday and multidose regimen raised concerns about adherence, e.g. in adolescents when taking medication at home with their parent(s), and since it cannot be directly observed. Treatment adherence is hard to assess, and pill intake may be overreported [140, 141]. However, the evidence implies that doxycycline is effective even with imperfect adherence and at lower doses than typically used [122, 141, 142]. It is important to counsel patients to avoid sex until they and their partner(s) have completed treatment. Additionally, there are suggestions that taking a daily dose of doxycycline may increase awareness and prevent people from resuming sex too early following treatment. Side effects include minor gastrointestinal upset, and photosensitivity may occur for doxycycline, although it is more common with longer or higher dosages and prevented by clear patient instructions [143]. In women, a limitation of doxycycline is that it is contraindicated in pregnancy. Doxycycline is an effective semisynthetic derivative in the class of tetracyclines. This class presents a documented teratogenic risk to the foetus, especially during the second trimester of pregnancy. These associations have also been applied to doxycycline but without evidence of teratogenicity for doxycycline during pregnancy; in fact, there are increasing reports that the use of doxycycline during the first trimester is not associated with teratogenicity during pregnancy, permanent tooth staining in pregnancy, hepatotoxicity, or permanent inhibitory bone growth effects [144]. Comparisons between azithromycin and doxycycline on side effects indicated conflicting results. No difference between azithromycin and doxycycline was observed in one meta-analysis [145]. Another systematic review found that the risk of side effects was lower in azithromycin in a mixed population of men and women, but no difference was noted in studies that only included men [3]. A study on MSM demonstrated that diarrhoea occurred more often with  azithromycin (40%) than with doxycycline (26%) use [127]. Taken together, while doxycycline might not be indicated in all situations or in all patients, overall doxycycline use should be considered widely applicable in women and in MSM.

*Shifting treatment activities in women and MSM* In both groups, most international guidelines include doxycycline as the first choice in rectal CT. The 2018 UK guidelines shifted from azithromycin toward doxycycline as a first-line treatment in non-pregnant women and in men in all anatomic sites, not only because of (at the time assumed) lower effectiveness in rectal CT, but also because of the expected azithromycin-AMR in non-CT pathogens. This shift was also prominent in the 2021 CDC guidelines [8], and at the moment of writing this paper, other international guidelines are in the process of collating all the evidence to update guidance.

A shift toward doxycycline does not prevent a possible treatment-induced, hampered immune response or a compromised microbiome, and it is also associated with health care costs. The main reason behind using the most effective treatment is to optimize the benefits of CT control activities (Fig. 1). It is assumed that timely treatment avoids symptom onset and progression into complications. However, the body of evidence is lacking for impact on the population level and is weak for impact on the individual level, as explained in “Test implementation in ‘real-life’ does not achieve the desired benefits” section. The desired benefit further includes reducing transmission to a partner, and additionally in women, preventing autoinoculation and subsequent vaginal infection [113]. A previous modelling study in women attending STI clinics assessed the expected reduction in population prevalence, taking into account rectal transmission and autoinoculation from the urogenital to the rectal site, and vice versa [31]. It estimated the impact of extending rectal testing from selective to universal, and of extending doxycycline use in rectal CT only to universal use. However, the extended scenarios only gave a very small additional reduction in CT prevalence in the models by 0.7–2% in 10 years (given an initial prevalence of 15%) compared to a scenario of selective rectal testing and doxycycline in rectal CT [31]. Nevertheless, for the individual patient and partners, when the goal is to prevent onward transmission to a partner or from the rectal to the vaginal site and according to current guidance, the most effective treatment should be the first choice. In women, the use of doxycycline in urogenital CT enables concurrent treatment of a large proportion of all rectal CT, even without rectal testing. Given that single-site rectal CT in women is uncommon (1–2% of all women at STI clinics) and single anatomic site CT more often spontaneously clears after diagnosis, rectal testing in women who are routinely urogenitally tested and treated with doxycycline, is not expected to yield important additional public health and clinical impacts. Future models might assess such single-site urogenital testing with doxycycline use in women, including new insights on viability, treatment failure, or autoinoculation. Future models might also evaluate single-site rectal testing in MSM since the rectal site is the main site of CT and CT-LGV infection in MSM. What is lacking are evaluations that assess both the realistically achievable benefits, especially complications prevented, and harms, including social, medical, and economic issues [67]. Increasingly, scientists are urging for more consideration of all these aspects, rethinking the net gains of testing and treatment, and then choosing the activities and activity levels, accordingly to reach these gains [11, 67].

## Conclusions

**Fig. 3:**
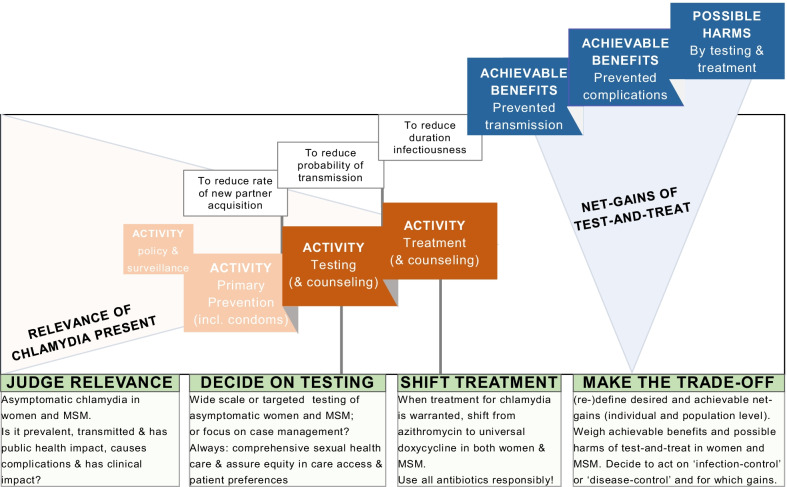
Key issues that need to be accounted for when choosing activities in testing and treating asymptomatic people in the context of the chlamydia control cascade

Test-and-treat has long been considered the cornerstone in chlamydia control, and various perspectives co-exist on what might be the best activities in asymptomatic women and MSM. The prevailing view on testing as many asymptomatic people as possible, especially young women, for urogenital CT is increasingly challenged by arguments to reduce testing efforts. Opposing views in extragenital CT testing are to test more to reveal missed rectal and pharyngeal CT in asymptomatic women and MSM, versus to stop testing for CT infections that have low or unknown relevance. Opposing perspectives in CT treatment are to use azithromycin versus doxycycline. Of note, the test and treatment of symptomatic people is not under debate. This paper addressed the recent controversies and highlighted state-of-the-art scientific studies on the claims raised. Here, we summarise the available evidence and assess whether controversies can now be resolved (Fig. 3).

### Controversies on testing

#### Women

*Urogenital testing in women.* In asymptomatic women, urogenital testing is routinely practised in STI clinics (CT control activity level iii, Fig. 1) and in some countries in primary care and the community (level iv). The prevalence of urogenital CT is substantial but highest in young women, with considerable variation by geography, sociodemographics, and test settings. New data indicate that STI clinic diagnosed women have viable vaginal CT and substantial organism loads. While CT is easily transmitted, the evidence on complication risks is more uncertain.

The assumed benefits of CT control activities are prevented transmission and complications. While mathematical models reveal the impact on transmission in theory, testing implementations in real life failed to realise reductions in prevalence and in PID and TFI at the population level. There is low to moderate evidence that testing can reduce PID risk at the individual level. However, the overall lack of evidence that assumed benefits of testing are realistically achievable raises questions on what the desired and achievable net gains are by pursuing widespread testing of asymptomatic people.

*Rectal testing in women.* Rectal CT testing is either discouraged or based on selective criteria, leaving a large share of all rectal CT undetected. The positivity of rectal CT, rectal CT viability, and rectal CT load is lower than that for urogenital CT, but still substantial in women tested at STI clinics and similar to that in MSM. Emerging evidence confirms that rectal CT in women likely represents ‘true' infections in several (though possibly not all) rectal CT cases. Such infections are potentially ‘transmissible’, although scientific data are lacking on female-to-male rectal CT transmission. Additionally, while observational studies and modelling data suggest transmission by autoinoculation, such a process is difficult to prove in humans. Rectal CT is largely asymptomatic and without later rectal complications; rectal CT may possibly contribute, via migration to the vaginal site, to reproductive complications, but this is speculative and unquantified.

*Pharyngeal testing in women.* Pharyngeal CT is infrequent. The public health and clinical impact of pharyngeal CT is nearly absent, and there are no known benefits of pharyngeal testing.

#### MSM

*Urogenital testing in MSM.* In MSM, urogenital CT is routinely tested, and even more frequently so in PrEP cohorts, as is anorectal and pharyngeal testing. However, the positivity in tested populations of urogenital CT is low, and urogenital LGV-CT is uncommon. The evidence for the benefits of testing asymptomatic MSM for urogenital CT is limited. While penile-anal sex is implicated as the main transmission route, urogenital CT is mostly asymptomatic and without complications.

*Rectal testing in MSM.* Rectal CT is routinely tested and most CT infections in MSM are at the rectal site. In some cases, this is the LGV biovar, resulting more often in symptoms and requiring a different treatment. Testing enables early treatment to prevent onward transmission, and to prevent the disease from developing in the individual, assuming treatment occurs in time. This is the basis for case management at the patient level and CT control activities in high-risk MSM cohorts. At the population level, however, the implementation of extensive (re)testing has not shown evidence that transmission can be substantially reduced. The main benefit of testing asymptomatic MSM for rectal CT is timely treatment of the LGV to prevent onward transmission and complications in the individual patient. Further, CT testing is an important point of entry for PrEP initiation and risk reduction counselling. In many countries, however, LGV typing is not routinely performed, except in symptomatic rectal CT in MSM. An indirect benefit at the population level is to help to prevent HIV transmission in populations with a high proportion of MSM not taking PrEP, although the evidence to support such an impact is low.

*Pharyngeal testing in MSM.* In MSM, routine pharyngeal testing is implemented by some countries. Pharyngeal CT has low positivity, low bacterial load, clears quickly and spontaneously, and does not importantly contribute to CT transmission. In asymptomatic MSM, the clinical benefits of testing urogenital or pharyngeal CT are minimal or have not been demonstrated.

### Controversies on treatment

*In both women and MSM*, azithromycin has long been the first line of treatment in CT and many other infectious diseases because it is a one-dose regimen that makes this treatment extremely applicable. In recent years, increasing reluctance has emerged to use azithromycin. The UK CT control guidelines were the first to recommend universal doxycycline use in CT, followed by the US CDC CT control guidelines, regardless of sex or the anatomic site of infection. This recommendation was primarily made due to increasing selection for macrolide resistance in co-present—but untested—non-CT (STI and other) pathogenic microorganisms.

Rectal CT is common in both women and MSM. Until recently, treatment effectiveness data in rectal CT were only available from observational studies. Two controlled studies in MSM were reported in 2021, indicating that azithromycin is inadequate for treating rectal CT. Doxycycline is highly effective in eradicating CT at all anatomic sites and in both sexes. The applicability of doxycycline has been noted as a potential problem in some contexts due to adherence issues and a contraindication in pregnancy. However, evidence suggests that doxycycline is likely effective even with shorter durations and lower doses, and can be safely used in women who cannot rule out early pregnancy, thus increasing the reach of this treatment strategy. Calls for responsible antibiotic use for any antibiotic because of possible harm, including AMR, are also important.

### Controversies resolved?

The controversy regarding treatment concerns the effectiveness, the possibility for widespread use, and harm of the main CT treatment regimens. Controlled studies provide much needed evidence to narrow the knowledge gap on effectiveness. In MSM and in women, evidence supports doxycycline as a first-line treatment in rectal CT. In women, evidence also supports its use in urogenital CT to appropriately treat concurrent—yet often untested—rectal CT. In women, using doxycycline in urogenital CT would co-treat most rectal CT in women without having to test all women rectally. In the recent international guidelines, AMR risk is weighted as crucial, and doxycycline is the preferred treatment in women and MSM irrespective of anatomic site. As is happening internationally, guidance is shifting toward using doxycycline universally Now, practice faces the challenge of implementation. Care professionals may be supported by sharing best practices that help to realise this shift in practice.

The controversies regarding testing are grounded in how we judge the ‘public health and clinical relevance’ of asymptomatic CT at a certain anatomic site and in a certain population. And in how we subsequently weigh the realistically achievable benefits versus the harms of testing and treating asymptomatic women and MSM. What are the net gains we want to strive for in CT control? (Fig. 3).

The available data urge us to more objectively address the relevance of asymptomatic CT; is it prevalent in the population of interest, is it important in onward transmission and has public health impact, and does it cause complications or otherwise have a clinical impact? Urogenital CT in women reveals an association with complications, although actual risks and preventable infections are uncertain. Infections that seem to have low clinical impact  include pharyngeal CT in women and MSM, and include asymptomatic urogenital CT in MSM. Rectal CT in women is mostly asymptomatic and a possible role of rectal CT in women on reproductive complications is speculative and unquantified. In MSM, rectal CT also is largely asymptomatic but may pose a risk for the spread of CT-LGV; although the impact on HIV transmission in the MSM population may be limited.

We need to set *realistic goals* for what are the desired and achievable benefits to test and treat these infections. The limitation of having very low to low evidence across most benefits complicates this discussion, and currently available studies do not provide insight into whether or how to target testing, or which CT control activity level [ii, iii or iv] is helpful. It is unknown where the benefits, if there are benefits, may be realised at the individual and population levels. Nevertheless, we should try and make this trade-off by balancing out possible hazards. This would require a (re-)definition of the net gains we are striving for  the individual or for the population. This is not an easy task. Possible sociopsychological harms and individual and societal costs (‘value for money’) need to be accounted for. Economic costs can be objectively calculated, but social and psychological aspects of testing are much more difficult to quantify. Additionally, the extent to which treatment of asymptomatic CT or non-viable CT will influence immunological reactions and affect AMR and microbiome issues is uncertain and not covered in current implementation decisions or in cost-effectiveness analyses. Although it is difficult to grasp the full impact of all possible harms related to treatment, the consensus is to limit antimicrobial use whenever possible, especially when there is AMR risk. Using antimicrobial treatment is a choice that should be made responsibly. The optimal application of (any) antimicrobial treatments in CT, while accounting for the broader social-medical and economic contexts, should be a topic for future debate.

Based on these considerations, we need to choose CT control activities to fit the realistic net-gains and we need to determine under which umbrella, i.e. infection control or disease control, we will take these steps. It is observed in some countries that CT control activities remain focused on infection control in the population, such as community-based testing of asymptomatic young women, targeted testing in asymptomatic key populations/high-risk venues, or the increasing STI testing efforts that accompany PrEP use in MSM. Alternatively, activities could more focus on disease control, to be more targeted on the individual patient and her/his partner via case management with appropriate diagnostic-clinic-partner services. This also includes the choice to limit or even stop testing asymptomatic persons altogether. As there is uncertainty about the benefits of widescale testing, and achievable benefits are likely smaller than assumed, and there also are harms, the question to date is whether we should test and treat all asymptomatic urogenital and extragenital infections in women and in MSM. The plea for a paradigm shift from infection control to disease control certainly applies to date, to focus more on health outcomes and to improve case management to prevent reinfections and complications. While these ideas are gaining ground in the literature and practice, whether and how the reduction of testing asymptomatics is translating into implementation and practice is not straightforward.

What decision will be made also depends on other factors, such as broader sexual health considerations. An offer for STI-testing can serve to link key populations to broader prevention and sexual health care services. Not pursuing CT testing could imply an opportunity lost to link key populations, such as young women, to comprehensive sexual health care. Additionally, in laboratory testing procedures, multiple STIs, such as CT and NG, are tested simultaneously. For example, NG has a different epidemiology, a different distribution over anatomic sites, and a different treatment, than CT; testing pharyngeal NG in MSM, for example, may have prominent public health and clinical benefits. Reflections in this paper only apply to CT.

We need to accept that there is no one-size-fits-all-‘best-strategy’. The judging of relevance of CT infections, the need to treat these and with what regimen, and the weighing of benefits and harms can differ between and within groups of patients, providers, and policies, even within a single country. Future studies that address these issues are needed to inform policy and practice. Thereby, issues related to equity, patient-and-provider preferences, acceptability, implementation, feasibility, and costs/resources, as well as broader sexual health, all need to be included.

### Limitations

This report does not specifically highlight important key populations such as transgender women, pregnant women, or HIV-infected people. However, the detailed overview provided enables researchers and professionals to make some inferences. A topic only minimally discussed is how to improve case management, such as retesting, using innovative ‘quick and easy’ methods for testing, expedited partner treatment, counselling and partner-notification strategies on e.g., whether it is needed to assume infection with rectal CT in women, concrete outreach approaches to target sexual partners, and smaller high-risk social networks. In general, all these methods could potentially generate benefits, yet all the concerns expressed in this paper likewise apply. Finally, although the literature was extensively searched and studies carefully evaluated and chosen to be included by all authors, this was not a systematic review, and the list of studies may not be exhaustive. However, to the best of our knowledge, all pertinent topics in this area were addressed and substantiated by a wide range of key relevant papers.

### Remaining knowledge gaps and interpretation

In recent years, CT research has greatly advanced our knowledge of CT epidemiology in terms of various populations and anatomic sites, including viability, bacterial load, and clearance, and risks for onward transmission (public health impact) or developing complications (clinical impact). Research has also demonstrated uncertainty regarding the benefits of testing asymptomatic women and MSM, and has highlighted possible harm. There are still knowledge gaps regarding why test-and-treat strategies do not work in real life as predicted in models. Reasons why test-and-treat strategies might not reduce prevalence are largely unknown but may include low test uptake, possible lower protective immunity in treated patients, and suboptimal sexual health care, including suboptimal treatment, e.g., azithromycin, and suboptimal case management, e.g., sexual partner notification, counselling on condom use, treatment compliance. The achievable benefits of widescale testing of asymptomatic women and MSM are likely much smaller than wished for, and the net gains in terms of outcomes need to be (re)defined for women and for MSM. This may shift the balance away from testing and treating asymptomatic persons. However, future evaluations are needed to better be able to make trade-offs, especially for urogenital CT in women (where the potential benefits of testing may be highest) and for rectal CT in MSM (due to CT-LGV and possible HIV risk in some settings). Care practice will need to shift towards implementing doxycycline in the treatment of CT, which may be supported by sharing best practices with challenges and opportunities in making the shift. We need future evaluations on CT control that address social, medical, and economic outcomes, assurances of equity in health care access, and which account for the target population’s preferences. The debate on the best strategies in CT control is gaining momentum, acknowledging diverse needs in numerous settings and populations.

## Supplementary Information

**Additional file 1.** Search strategy in Pubmed Central

## Data Availability

Data sharing is not applicable to this article as no datasets were generated or analysed during the current study.

## Abbreviations

AMR: Anti microbial resistance
CT: *Chlamydia trachomatis*
DNA: Deoxyribonucleic acid
EP: Ectopic pregnancy
HIV: Human immunodeficiency virus
LGV: Lymphogranuloma venereum
MG: *Mycoplasma genitalium*
MSM: Men who have sex with men
NAAT: Nucleic acid amplification test
NG: *Neisseria gonorrhoeae*
PID: Pelvic inflammatory disease
PrEP: Pre-exposure prophylaxis
RNA: Ribonucleic acid
STI: Sexually transmitted infections
TFI: Tubal factor infertility
V-PCR: Viability polymerase chain reaction assay
