# Supplementary material for: Controversies and evidence on Chlamydia testing and treatment in asymptomatic women and men who have sex with men: a narrative review
Source: BMC Infect Dis. 2022 Mar 14;22:255. doi: 10.1186/s12879-022-07171-2 (PMC8922931; doi:10.1186/s12879-022-07171-2)
Supplement: Supplementary file 1 — Additional file 1. Search strategy in Pubmed Central [file 12879_2022_7171_MOESM1_ESM.docx]

**Supplementary file I: Search strategy in Pubmed Central**

*Prevalence or positivity or testing or screening or transmission or viability or load or treatment or rectal or anorectal or anorectum or anal or pharyngeal or oropharyngeal or oropharynx or oral or pelvic inflammatory disease or PID or Complications or anatomical site*

*And Chlamydia*

*And Women or female or men who have sex with men or MSM*

*And ("2018/01/01"[PubDate] : "2021/09/30"[PubDate])*
